# Individual and Combined Expression of DNA Damage Response Molecules PARP1, γH2AX, BRCA1, and BRCA2 Predict Shorter Survival of Soft Tissue Sarcoma Patients

**DOI:** 10.1371/journal.pone.0163193

**Published:** 2016-09-19

**Authors:** Kyoung Min Kim, Young Jae Moon, See-Hyoung Park, Hye Jeong Park, Sung Il Wang, Ho Sung Park, Ho Lee, Keun Sang Kwon, Woo Sung Moon, Dong Geun Lee, Jung Ryul Kim, Kyu Yun Jang

**Affiliations:** 1 Department of Pathology, Chonbuk National University Medical School, Research Institute of Clinical Medicine of Chonbuk National University-Biomedical Research Institute of Chonbuk National University Hospital and Research Institute for Endocrine Sciences, Jeonju, Republic of Korea; 2 Department of Orthopedic Surgery, Chonbuk National University Medical School, Research Institute of Clinical Medicine of Chonbuk National University-Biomedical Research Institute of Chonbuk National University Hospital and Research Institute for Endocrine Sciences, Jeonju, Republic of Korea; 3 Department of Bio and Chemical Engineering, Hongik University, Sejong, Republic of Korea; 4 Department of Forensic Medicine, Chonbuk National University Medical School, Jeonju, Republic of Korea; 5 Department of Preventive Medicine, Chonbuk National University Medical School, Jeonju, Republic of Korea; University of Pittsburgh Cancer Institute, UNITED STATES

## Abstract

DNA damage response (DDR) molecules are protective against genotoxic stresses. DDR molecules are also involved in the survival of cancer cells in patients undergoing anti-cancer therapies. Therefore, DDR molecules are potential markers of cancer progression in addition to being potential therapeutic targets. In this study, we evaluated the immunohistochemical expression of PARP1, γH2AX, BRCA1, and BRCA2 and their prognostic significance in 112 cases of soft tissue sarcoma (STS). The expression of PARP1, γH2AX, BRCA1, and BRCA2 were significantly associated with each other and were associated with higher tumor stage and presence of distant metastasis. The expression of PARP1, γH2AX, and BRCA2 were significantly associated with shorter disease-specific survival (DSS) and event-free survival (EFS) by univariate analysis. BRCA1 expression was associated with shorter DSS. Multivariate analysis revealed the expression of PARP1 and γH2AX to be independent indicators of poor prognosis of DSS and EFS. BRCA2 expression was an independent indicator of poor prognosis of DSS. In addition, the combined expressional patterns of PARP1, γH2AX, BRCA1, and BRCA2 (CSddrm) were independent prognostic predictors of DSS (*P* < 0.001) and EFS (*P* = 0.016). The ten-year DSS rate of the CSddrm-low, CSddrm-intermediate, and CSddrm-high subgroups were 81%, 26%, and 0%, respectively. In conclusion, this study demonstrates that the individual and combined expression patterns of the DDR molecules PARP1, γH2AX, BRCA1, and BRCA2 could be predictive of the prognosis of STS patients and suggests that controlling the activity of these DDR molecules could be employed in new therapeutic stratagems for the treatment of STS.

## Introduction

An early event in response to a DNA single stand break (SSB) is immediate binding of poly ADP-ribose polymerase 1 (PARP1) to the DNA break, which then recruits other DNA repair proteins [1, 2]. If the repair of a DNA SSB is unsuccessful and the damage progresses to a DNA double strand break (DSB), phosphorylated H2AX (γH2AX) and BRCA1/2 are then recruited to repair the DNA DSB [3–5]. Defects in DNA damage repair mechanisms result in genomic instability and accumulation of additional mutations [6]. Therefore, the expression of DNA damage response (DDR) molecules are believed to limit the development of cancer [6]. Mutations or defects in *BRCA1/2* are closely related with early development of breast and ovarian carcinomas [7–9]. However, the concept of the tumor suppressive roles of DDR molecules has changed by engaging modern therapeutic modalities such as chemotherapeutic agents to induce DNA damage to promote apoptosis, and radiation therapy [1, 3, 4, 6]. DNA breaks induced by anti-cancer therapeutic modalities could be insufficient in inducing the death of tumor cells due to the repair function of DDR molecules [1, 3, 6]. In this context, the expression of DDR molecules during anti-cancer therapy could be associated with resistance to therapy [10–12]. Therefore, paradoxically, the expression of DDR molecules such as PARP1 and γH2AX could provide resistance to anti-cancer chemotherapy and radiation therapy, which induce cell death by causing DNA damage [4, 10, 11]. Moreover, it has been suggested that the expression of PARP1, γH2AX, BRCA1, and BRCA2 are closely associated with the progression of various human malignant tumors [3, 10, 13–18]. The expression of PARP1 [14, 15, 17–20] and γH2AX [13, 14, 21] were significantly associated with shorter survival of various human malignant tumors. It has been suggested that the expression of BRCA1/2 might also be related with chemoresistance and shorter survival of patients with breast carcinoma [14] and ovarian carcinoma [16]. Moreover, our previous study demonstrated that the combined expression pattern of PARP1, γH2AX, BRCA1, and BRCA2 is very helpful in the prediction of the prognosis of breast carcinoma [14].

Based on the rationale that DDR molecules are involved in the progression of cancers and resistance to the anti-cancer treatments, it has been suggested that DDR molecules could be therapeutic targets of malignant tumors, and therapy targeting DDR molecules is under evaluation [6, 20, 22, 23]. Inhibition of PARP1 was applied to the treatment of cancer patients in conjunction with conventional therapy inducing DNA damage [24, 25]. Especially, when there are defects in DSB repair by mutation of *BRCA1/2*, inhibition of PARP1 results in un-repairable DNA DSBs and apoptosis of cancer cells [1, 3, 23, 26, 27]. Although there are controversies, chemotherapeutic effectiveness of PARP1 inhibitors have been assessed in *BRCA*-deficient breast carcinomas [23, 27] and ovarian carcinomas with a *BRCA1/2* mutation [26].

Soft tissue sarcomas (STSs) are not a common type of malignant tumor and approximately 50 new STSs develop per million people annually [28]. The five-year survival rates of STS of children is variable, being reported as 36.3% in India to 72.1% in Australia [9]. In addition, reliable therapeutic application for STS is limited and surgery remains the principal therapy [28]. Therefore, during the evaluation of possible therapeutic target of STSs, we previously reported that the expression of SIRT1, deleted in breast cancer 1, β-catenin, programed death 1, and PD-L1 were closely associated with progression of STSs [29, 30]. Recently, the possibility that PARP1 inhibitors could have therapeutic efficacy for STSs has been suggested. The PARP1 inhibitor, olaparib, inhibited the proliferation of malignant peripheral nerve sheath tumor cells [31] and another PARP1 inhibitor, rucaparib, induced apoptosis and inhibited the proliferation of rhabdomyosarcoma cells [32]. In Ewing sarcoma, an EWS-FLI1 fusion gene induced PARP1 expression and the fusion gene also induced DNA damage, which was potentiated by a PARP1 inhibitor [33]. However, a single administration of olaparib did not achieve a therapeutic response in 12 recurrent Ewing sarcoma patients [34, 35]. Therefore, combined use of PARP1 inhibitors and conventional genotoxic cancer therapeutic agents has been suggested for the treatment of malignant tumors. Especially, combined use of a PARP inhibitor and a DNA damaging agent was suggested as a treatment for Ewing sarcoma because Ewing sarcoma has defect in the DNA break repair system [36]. A recent report has shown that combined use of PARP inhibitors (niraparib, rucaparib, olaparib, BMN-673, talazoparib, or veliparib) and temozolomide inhibited proliferation and induced apoptosis of Ewing sarcoma cells [24, 37]. In an EWS-FLI1 fusion gene-positive xenograft model in mice, combined use of olaparib and temozolomide induced a complete response [33]. Therefore, based on the possibility that DDR molecules might be therapeutic targets of STS, this study examined the immunohistochemical expression of the DDR molecules PARP1, γH2AX, BRCA1, and BRCA2 in STSs, and evaluated the prognostic significance of their expression in STSs.

## Materials and Methods

### Ethics

This study obtained institutional review board approval from Chonbuk National University Hospital and the requirement for informed consent was waived (IRB number, CUH 2013-07-036). All experiments were performed in accordance with relevant guidelines and regulations.

### Patients and samples

Primary STSs from therapeutic surgical resections between July 1998 and January 2013 at Chonbuk National University Hospital, including 105 cases of STSs included in our previous studies were subjects of this study [29, 30]. Thereafter, cases for which original histologic slides and paraffin blocks were available were evaluated for this study. The original histologic slides were reviewed and classified according to the 2013 World Health Organization classification of tumors of soft tissue and bone [28]. Thereafter, 112 cases of STSs other than gastrointestinal stromal tumors, Kaposi’s sarcomas, and atypical lipomatous tumors previously diagnosed as well-differentiated liposarcomas were included in this study. The histologic subtypes of STS included in this study are listed in Table 1. In addition to the resection of primary lesion of STS, forty-five patients received adjuvant chemotherapy; forty patients received radiation therapy; eighteen patients received both adjuvant chemotherapy and radiation therapy; and forty-five patients received no adjuvant treatment. Tumors were graded primarily according to the FNCLCC (French Fédération Nationale des Centres de Lutte Contre le Cancer) grading system [38] and WHO classification [28]. Tumor stage was based on the guidelines of the American Joint Committee on Cancer [39]. Clinical information was obtained by reviewing medical records.

**Table 1:** The expression status of PARP1, γH2AX, BRCA1, and BRCA2 according to the histological type of soft-tissue sarcoma.

Histological type	No.	PARP1	γH2AX	BRCA1	BRCA2
		positive	positive	positive	positive
Leiomyosarcoma	20	14 (70%)	13 (65%)	12 (60%)	15 (75%)
Synovial sarcoma	17	15 (88%)	11 (65%)	9 (53%)	14 (82%)
Undifferentiated sarcoma	12	8 (67%)	10 (83%)	6 (50%)	7 (58%)
Myxoid liposarcoma	10	1 (10%)	1 (10%)	2 (20%)	2 (20%)
Well differentiated liposarcoma	4	1 (25%)	0 (0%)	2 (50%)	1 (25%)
Dedifferentiated liposarcoma	3	0 (0%)	1 (33%)	0 (0%)	0 (0%)
Ewing sarcoma	6	3 (50%)	3 (50%)	2 (33%)	4 (67%)
Malignant peripheral nerve sheath tumor	6	2 (33%)	3 (50%)	1 (17%)	2 (33%)
Angiosarcoma	6	3 (50%)	5 (83%)	2 (33%)	5 (83%)
Myxofibrosarcoma	6	1 (17%)	2 (33%)	2 (33%)	0 (0%)
Adult fibrosarcoma	5	2 (40%)	3 (60%)	4 (80%)	1 (20%)
Epithelioid sarcoma	4	4 (100%)	3 (75%)	3 (75%)	1 (25%)
Low grade myofibroblastic sarcoma	4	1 (25%)	1 (25%)	1 (25%)	1 (25%)
Alveolar rhabdomyosarcoma	3	3 (100%)	3 (100%)	1 (33%)	2 (67%)
Embryonal rhabdomyosarcoma	2	2 (100%)	0 (0%)	0 (0%)	0 (0%)
Pleomorphic rhabdomyosarcoma	2	1 (50%)	1 (50%)	1 (50%)	1 (50%)
Spindle cell/sclerosing rhabdomyosarcoma	1	1 (100%)	1 (100)	1 (100%)	0 (0%)
Clear cell sarcoma	1	1 (100%)	0 (0%)	1 (100%)	1 (100%)

### Establishment of tissue microarray and immunohistochemical staining

Tissue microarrays (TMA) were established from original paraffin-embedded tissue blocks. To construct TMA, original H&E slides were reviewed and two 3.0 mm cores were taken from the most representative solid area composed of intact tumor cells with the highest histological grade. The TMA tissue sections underwent antigen retrieval by placing in Dako Target Retrieval Solution (pH 6.0, DAKO, Glostrup, Denmark) for 20 minutes in a microwave oven and incubated with primary antibodies for PARP1 (1:100, Santa Cruz Biotechnology, Santa Cruz, CA, USA), γH2AX (Ser 139) (1:100, Cell Signaling Technology, Beverly, MA, USA), BRCA1 (1:100, Abcam, Cambridge, MA, USA), and BRCA2 (1:100, Abcam, Cambridge, MA, USA). Immunohistochemical staining slides were evaluated by three pathologists (JKY, PHS and KKM) by consensus under a multi-viewing microscope without information of the clinicopathological factors. Because the principal functions of PARP1, BRCA1, and BRCA2 are related with their nuclear expression and previous reports have shown that their expression in the nucleus was associated with tumor progression [14, 16, 40, 41], we also evaluated the nuclear expression of PARP1, BRCA1, and BRCA2 in this study. Immunohistochemical expression of PARP1, BRCA1, and BRCA2 were evaluated by the sum of the staining intensity scores (0; no staining, 1; weak staining, 2; intermediate staining, and 3; strong staining) and the staining area scores (0; no staining cells, 1; 1% of the cells stained positive, 2; 2–10% of the cells stained positive, 3; 11–33% of the cells stained positive, 4; 34–66% of the cells stained positive, and 5; 67–100% of the cells stained positive) in each TMA core [14, 30, 42]. Thereafter, the score of two TMA cores from the same case were added and used for the analysis (sum score). The sum score ranged from zero to sixteen [14, 30]. To evaluate the immunohistochemical expression of γH2AX, the highest γH2AX-scoring areas were selected at low-power field and the number of γH2AX-positive tumor cells in five high-power fields (HFP, magnification; x400) were assessed in each TMA core. Thereafter, the final number of γH2AX-positive tumor cells in each case were obtained by adding the number of γH2AX-positive tumor cells from the two different TMA cores [14, 29, 43]. The diameter of the HPF was 0.55 mm and the area of one HPF was 0.238 mm^2^.

### Statistical analysis

Based on the immunohistochemical staining scores of the PARP1, γH2AX, BRCA1, and BRCA2 expression, the STSs were grouped as negative or positive for each stain. The cut-off points for the immunohistochemical staining scores of each marker were determined by receiver operating characteristic curve analysis. The points showing the highest area of under curve to estimate the death of patients were selected as the cut-off point for each marker. The endpoint of follow-up was the date of death of patients or the date of last contact through December 2013. The prognosis was evaluated by analyzing disease-specific survival (DSS) and event-free survival (EFS). The duration of DSS was calculated from the date of diagnosis to the date of death from STSs or the date of last contact. The patients who were alive at last contact or died from other causes were treated as censored. The duration of EFS was calculated from the date of diagnosis to the date of death from STS, the date of relapse, or the date of last contact. The patients who were alive without relapse at last contact or died from other causes were treated as censored for EFS analysis. Statistical analysis was performed using SPSS statistical software (IBM, version 20.0, CA, USA). The correlation between the clinicopathological factors that subjected in this study were evaluated by Pearson’s chi-square test and the *P* values were adjusted by Bonferroni correction for multiple comparison. The univariate and multivariate Cox regression hazard analysis and Kaplan-Meier survival analysis were performed for the survival analysis. *P*-values less than 0.05 were considered to be statistically significant.

## Results

### The association between the clinicopathological variables of STSs and the expression of PARP1, γH2AX, BRCA1, and BRCA2

**Fig 1:**
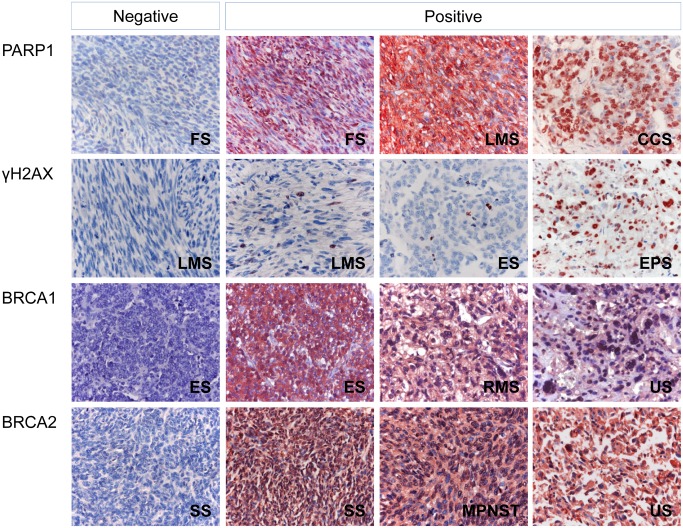
Immunohistochemical expression of PARP1, γH2AX, BRCA1, and BRCA2 in various soft tissue sarcomas. Abbreviations: FS, adult fibrosarcoma; LMS, leiomyosarcoma; MPNST, malignant peripheral nerve sheath tumor; ES, Ewing sarcoma; CCS; clear cell sarcoma; SS, synovial sarcoma; RMS, rhabdomyosarcoma; US, undifferentiated sarcoma. Original magnification, x400.

**Fig 2:**
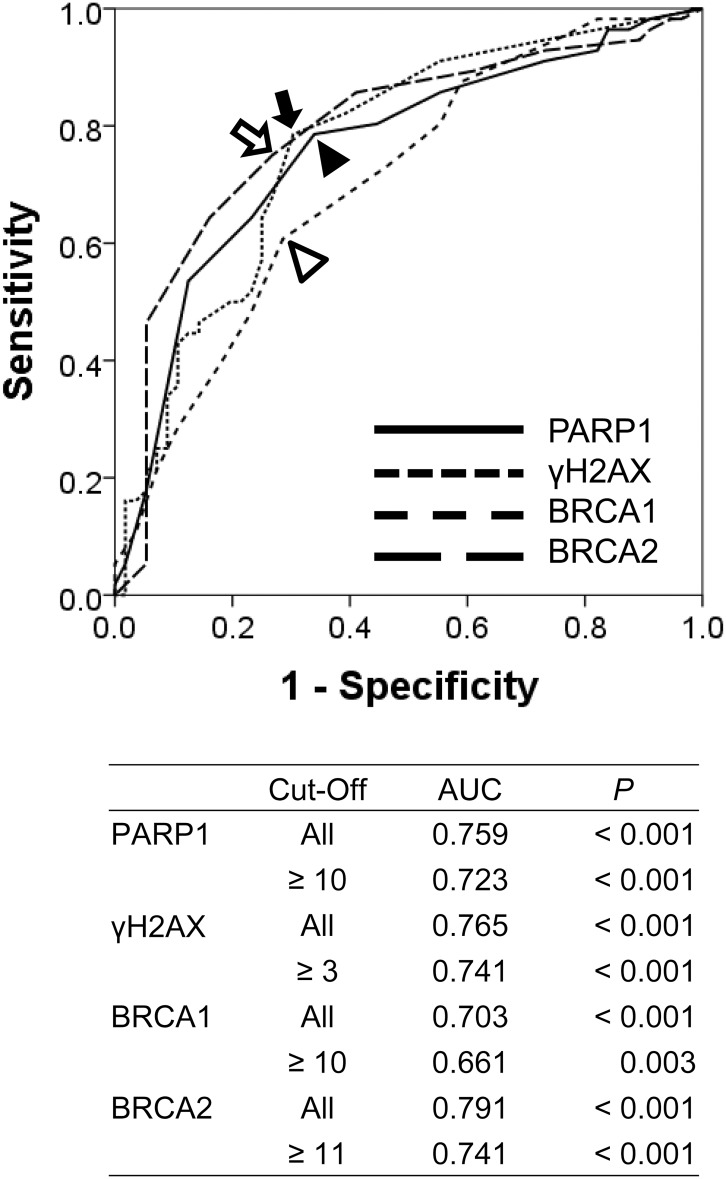
Statistical analysis to determine the cut-off points for the immunohistochemical staining scores of PARP1, γH2AX, BRCA1, and BRCA2. The cut-off points for the immunohistochemical staining of PARP1 (black arrow head), γH2AX (black arrow), BRCA1 (empty arrow head), and BRCA2 (empty arrow) were determined by the receiver operating characteristic curve analysis at the highest AUC (area under the curve) value for the estimation of the event of disease-specific survival of patients. Cases with scores equal to or greater than 10 for PARP1 and BRCA1 expression were considered positive. The expression of γH2AX was considered positive when the number of γH2AX-positive cells was equal to or greater than three. The expression of BRCA2 was considered positive when the scores were equal to or greater than 11.

As we have shown in Fig 1, γH2AX is primarily expressed in the nuclei of tumor cells. However, although we evaluated the nuclear expression of PARP1, BRCA1, and BRCA2 based on the previous reports [14, 16, 40, 41], the expression of PARP1, BRCA1, and BRCA2 are seen in both the cytoplasm and nuclei of tumor cells (Fig 1). The cut-off points for the immunohistochemical staining scores for the nuclear expressions of PARP1, BRCA1, and BRCA2 were 10, 10, and 11, respectively (Fig 2). Immunohistochemical stains were considered positive if scores were equal to or greater than 10 for PARP1 and BRCA1, and were considered positive if scores were equal to or greater than 11 for BRCA2. The cut-off point for the γH2AX immunohistochemical staining was three. Immunostaining for γH2AX was grouped as positive when there were more than three γH2AX-positive cells in 10 HPF from two TMA cores (Fig 2). When using these cut-off values, 56% (63 of 112 of cases), 54% (61 of 112 of cases), 45% (50 of 112 of cases), and 51% (57 of 112 of cases) of STSs were grouped as positive for PARP1, γH2AX, BRCA1, and BRCA2 staining, respectively. The positivity of PARP1, γH2AX, BRCA1, and BRCA2 expression varied according to the histologic subtypes of STSs (Table 1). In overall STSs, the expression of PARP1, γH2AX, and BRCA2 all correlated with aggressive tumor features, including advanced tumor stage and distant metastasis, while PARP1 and γH2AX correlated with poor histologic prognosticators, including higher histologic grade and increased mitotic count, as well as with expression of other DDR molecules (Table 2).

**Table 2:** The correlation between the clinicopathological variables and the expression of PARP1, γH2AX, BRCA1, and BRCA2 in soft tissue sarcomas.

Characteristics		No	PARP1		γH2AX		BRCA1		BRCA2	
			+	*P*_*B*_	+	*P*_*B*_	+	*P*_*B*_	+	*P*_*B*_
Age, y	< 60	69	39 (57%)	1.000	35 (51%)	0.995	28 (41%)	0.989	33 (48%)	0.999
	≥ 60	43	24 (56%)		26 (60%)		22 (51%)		24 (56%)	
Sex	female	48	25 (52%)	1.000	28 (58%)	0.999	19 (40%)	0.998	28 (58%)	0.930
	male	64	38 (59%)		33 (52%)		31 (48%)		29 (45%)	
Stage	I and II	55	22 (40%)	0.009	21 (38%)	0.009	17 (31%)	0.056	18 (33%)	0.002
	III and IV	57	41 (72%)		40 (70%)		33 (58%)		39 (68%)	
Depth	superficial	41	17 (41%)	0.208	14 (34%)	0.014	13 (32%)	0.405	14 (34%)	0.094
	deep	71	46 (65%)		47 (66%)		37 (52%)		43 (61%)	
Tumor size, cm	≤ 5	38	19 (50%)	0.997	19 (50%)	0.999	17 (45%)	1.000	18 (47%)	1.000
	> 5	74	44 (59%)		42 (57%)		33 (45%)		39 (53%)	
LN metastasis	absence	95	49 (52%)	0.230	49 (52%)	0.892	42 (44%)	1.000	48 (51%)	1.000
	presence	17	14 (82%)		12 (71%)		8 (47%)		9 (53%)	
Distant metastasis	absence	81	38 (47%)	0.018	36 (44%)	0.008	31 (38%)	0.331	32 (40%)	0.001
	presence	31	25 (81%)		25 (81%)		19 (61%)		25 (81%)	
Histological Grade	1	24	5 (21%)	0.003	4 (17%)	< 0.001	8 (33%)	0.979	6 (25%)	0.068
	2	39	23 (59%)		20 (51%)		16 (41%)		19 (49%)	
	3	49	35 (71%)		37 (76%)		26 (53%)		32 (65%)	
Tumor differentiation	1	11	4 (36%)	0.034	3 (27%)	0.194	7 (64%)	0.978	4 (36%)	0.651
	2	46	19 (41%)		21 (46%)		17 (37%)		19 (41%)	
	3	55	40 (73%)		37 (67%)		26 (47%)		34 (62%)	
Mitotic count	0-9/10 HPF	44	16 (36%)	0.021	13 (30%)	0.001	16 (36%)	0.998	16 (36%)	0.464
	10-19/10 HPF	22	13 (59%)		14 (64%)		11 (50%)		14 (64%)	
	> 19/10 HPF	46	34 (74%)		34 (74%)		23 (50%)		27 (59%)	
Tumor necrosis	no necrosis	55	28 (51%)	1.000	20 (36%)	0.009	22 (40%)	1.000	21 (38%)	0.284
	< 50%	43	26 (60%)		30 (70%)		20 (47%)		26 (60%)	
	≥ 50%	14	9 (64%)		11 (79%)		8 (57%)		10 (71%)	
BRCA2	negative	55	21 (38%)	0.002	19 (35%)	< 0.001	13 (24%)	< 0.001		
	positive	57	42 (74%)		42 (74%)		37 (65%)			
BRCA1	negative	62	26 (42%)	0.009	28 (45%)	0.325				
	positive	50	37 (74%)		33 (66%)					
γH2AX	negative	51	21 (41%)	0.045						
	positive	61	42 (69%)							

*P*_*B*_; Chi-square test adjusted by Bonferroni correction.

### The expression of PARP1, γH2AX, BRCA1, and BRCA2 were associated with shorter survival of STS patients by univariate analysis

**Fig 3:**
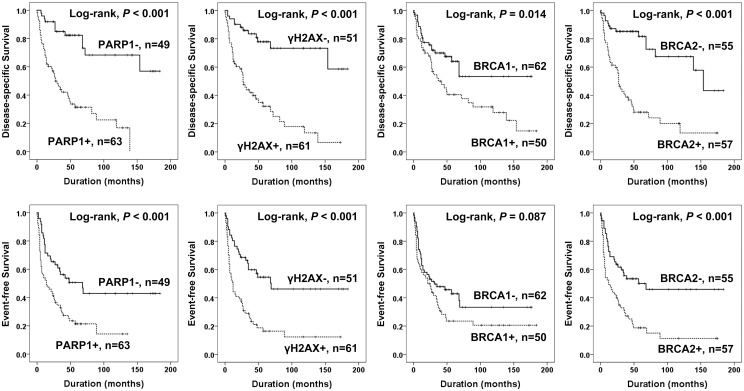
Kaplan-Meier survival analysis in soft tissue sarcomas. Disease-specific survival and event-free survival according to the expression of PARP1, γH2AX, BRCA1, and BRCA2.

**Fig 4:**
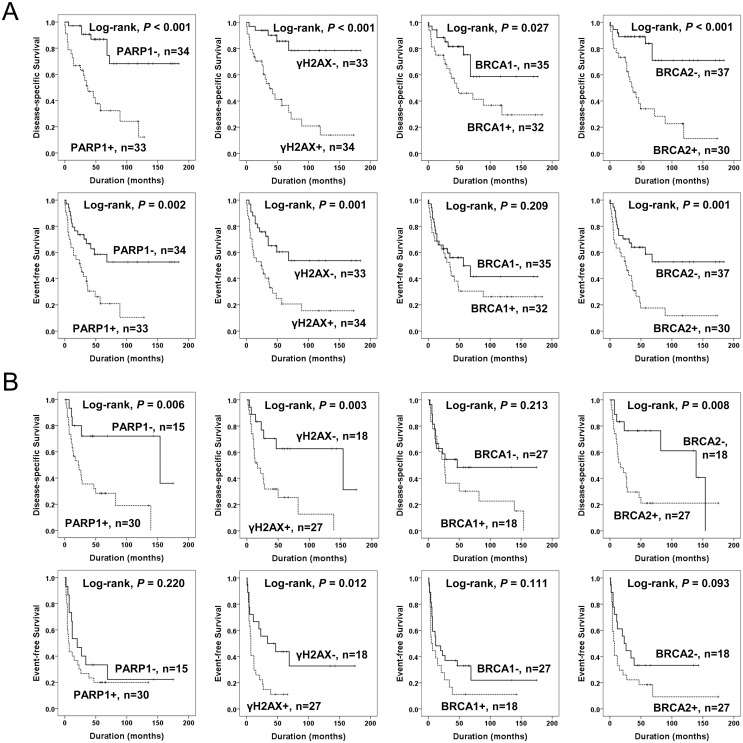
Kaplan-Meier survival analysis of the sub-populations of soft tissue sarcomas according to adjuvant chemotherapy. Disease-specific survival and event-free survival according to the expression of PARP1, γH2AX, BRCA1, and BRCA2 in 67 soft tissue sarcoma patients who did not received adjuvant chemotherapy (A) and 45 soft tissue sarcoma patients who received adjuvant chemotherapy (B).

**Fig 5:**
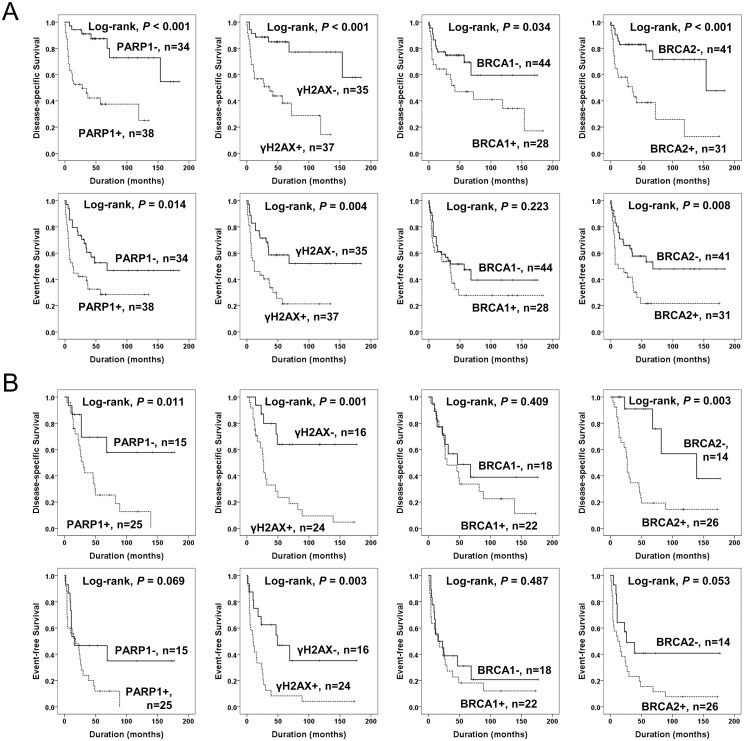
Kaplan-Meier survival analysis of the sub-populations of soft tissue sarcomas according to adjuvant radiotherapy. Disease-specific survival and event-free survival according to the expression of PARP1, γH2AX, BRCA1, and BRCA2 in 72 soft tissue sarcoma patients who did not received adjuvant radiotherapy (A) and 40 soft tissue sarcoma patients who received adjuvant radiotherapy (B).

**Fig 6:**
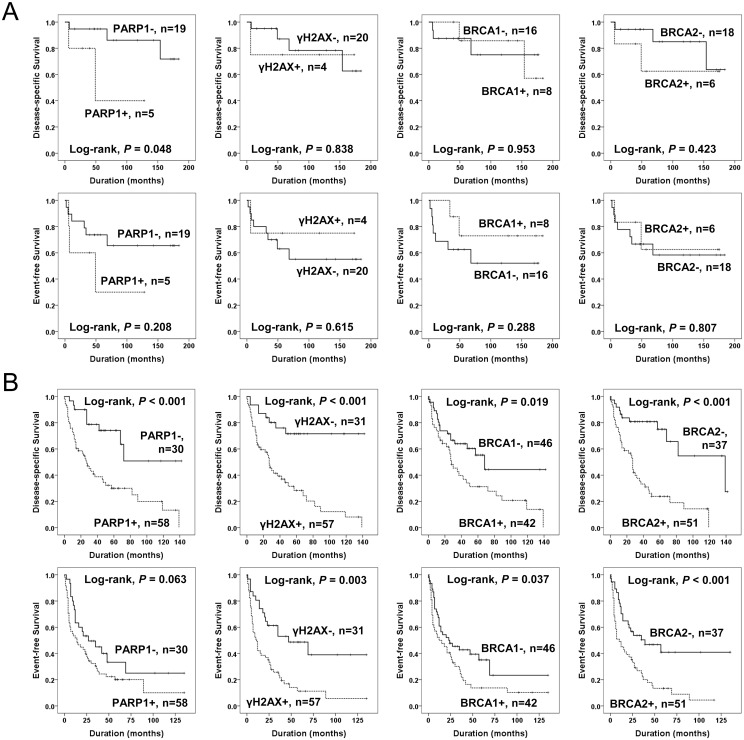
Kaplan-Meier survival analysis of the sub-populations of soft tissue sarcomas according to grade. Disease-specific survival and event-free survival according to the expression of PARP1, γH2AX, BRCA1, and BRCA2 in 24 low grade soft tissue sarcoma patients (A) and 88 high grade soft tissue sarcoma patients (B).

The factors significantly associated with both shorter DSS and EFS in 112 cases of STSs were the age of the patients, tumor stage, depth of tumor, lymph node metastasis, distant metastasis, histologic grade, the number of cells undergoing mitosis, tumor necrosis, and the expression of PARP1, γH2AX, and BRCA2 (Fig 3). The expression of PARP1 predicted a 5.021-fold greater risk of death and a 2.239-fold greater risk of death or relapse of STS patients. The patients having γH2AX-positive tumors had a 4.928-fold greater risk of death and γH2AX expression was significantly associated with shorter EFS. The expression of BRCA2 predicted shorter DSS and EFS. The expression of BRCA1 was associated with shorter DSS but not with shorter EFS (Table 3). Subsequently, we performed further survival analysis in the subpopulation of STS patients who received adjuvant chemotherapy or radiotherapy. The expression of PARP1, γH2AX, and BRCA2 were associated with shorter survival in both the subgroup of STS patients who received adjuvant chemotherapy and those that did not (Fig 4). In addition, the expression of PARP1, γH2AX, and BRCA2 were associated with shorter survival in both the subgroup of patients who received adjuvant radiotherapy and those that did not (Fig 5). Because biologic behaviors are significantly different between low grade and high grade (grade 2 and 3) STSs, we performed additional Kaplan-Meier survival analysis in both low grade and high grade STSs. In the subpopulation of low grade STSs, only the expression of PARP1 was significantly associated with shorter DSS (Fig 6A). However, in the subpopulation of high grade STSs, the expression of PARP1, γH2AX, BRCA1, and BRCA2 were significantly associated with shorter DSS, and the expression of γH2AX, BRCA1, and BRCA2 were significantly associated with shorter EFS (Fig 6B). In addition, when further analysis was performed according to the histologic subtypes of STSs (Table 4), PARP1 expression was significantly associated with shorter survival of leiomyosarcoma, myxofibrosarcoma, and adult fibrosarcoma. γH2AX-positivity was significantly associated with shorter EFS in synovial sarcoma and myxofibrosarcoma. BRCA1 expression was associated with shorter DSS in synovial sarcoma and shorter EFS in malignant peripheral nerve sheath tumor. BRCA2 expression was associated with shorter survival in malignant peripheral nerve sheath tumors.

**Table 3:** Univariate Cox regression analysis for disease-specific survival and event-free survival in soft-tissue sarcoma patients.

Characteristics	No.	DSS		EFS	
		HR (95% CI)	*P*	HR (95% CI)	*P*
Age, y, ≥ 60 (*vs* < 60)	43/112	1.826 (1.076–3.099)	0.026	2.010 (1.268–3.187)	0.003
Sex, male (*vs* female)	64/112	1.496 (0.869–2.576)	0.146	1.232 (0.775–1.961)	0.378
Stage, III & IV (*vs* I & II)	57/112	4.536 (2.430–8.468)	< 0.001	2.587 (1.604–4.170)	< 0.001
Depth, deep (*vs* superficial)	71/112	3.915 (1.916–7.999)	< 0.001	2.984 (1.730–5.145)	< 0.001
Size, cm, > 5 (*vs* ≤ 5)	74/112	1.534 (0.848–2.776)	0.157	1.043 (0.644–1.690)	0.864
LN metastasis, + (*vs* -)	17/112	2.469 (1.323–4.608)	0.005	1.882 (1.048–3.378)	0.034
Distant metastasis, + (*vs* -)	31/112	4.170 (2.442–7.122)	< 0.001	3.607 (2.235–5.824)	< 0.001
Grade, 1	24/112	1	< 0.001	1	0.003
2	39/112	4.648 (1.556–13.890)	0.006	2.795 (1.305–5.987)	0.008
3	49/112	7.392 (2.549–21.440)	< 0.001	3.603 (1.722–7.536)	< 0.001
Differentiation, 1	11/112	1	0.041	1	0.159
2	46/112	2.414 (0.717–8.124)	0.155	1.423 (0.589–3.440)	0.433
3	55/112	3.864 (1.166–12.805)	0.027	2.021 (0.854–4.785)	0.11
Mitotic count, 0–9	44/112	1	0.001	1	0.005
10–19	22/112	3.294 (1.502–7.223)	0.003	2.366 (1.247–4.487)	0.008
> 19	46/112	3.486 (1.754–6.929)	< 0.001	2.313 (1.338–3.999)	0.003
Necrosis, no	55/112	1	< 0.001	1	0.027
< 50%	43/112	3.277 (1.778–6.041)	< 0.001	1.947 (1.187–3.193)	0.008
≥ 50%	14/112	3.655 (1.642–8.133)	0.001	1.709 (0.833–3.504)	0.144
PARP1, + (*vs* -)	63/112	5.021 (2.564–9.830)	< 0.001	2.239 (1.377–3.641)	0.001
γH2AX, + (*vs* -)	61/112	4.928 (2.581–9.408)	< 0.001	2.784 (1.700–4.559)	< 0.001
BRCA1, + (*vs* -)	50/112	1.932 (1.126–3.314)	0.017	1.479 (0.937–2.336)	0.093
BRCA2, + (*vs* -)	57/112	4.289 (2.329–7.901)	< 0.001	2.403 (1.493–3.868)	< 0.001
CSddrm, negative (score 0–1)	45/112	1	< 0.001	1	< 0.001
low (score 2–3)	45/112	7.858 (3.252–18.986)	< 0.001	2.372 (1.362–4.128)	0.002
high (score 4)	22/112	13.958 (5.452–35.738)	< 0.001	3.970 (2.114–7.457)	< 0.001

Abbreviations: DSS; disease-specific survival, EFS; event-free survival, HR; hazard ratio, 95% CI; 95% confidence interval, CSddrm; the combined score for the immunohistochemical expression of PARP1, γH2AX, BRCA1, and BRCA2.

**Table 4:** Kaplan-Meier survival analysis with Log-rank test according to the expressions of PARP1, γH2AX, BRCA1, and BRCA2 in various histological types of soft-tissue sarcomas.

Histologic type	No.	DSS (Log-rank, *P*)					EFS (Log-rank, *P*)				
		PARP1	γH2AX	BRCA1	BRCA2	CSddrm	PARP1	γH2AX	BRCA1	BRCA2	CSddrm
Leiomyosarcoma	20	0.008	0.296	0.408	0.122	0.200	0.010	0.677	0.390	0.143	0.445
Synovial sarcoma	17	0.209	0.103	0.016	0.065	0.008	0.838	0.036	0.060	0.051	0.019
Undifferentiated sarcoma	12	0.612	0.612	0.843	0.282	0.435	0.546	0.9	0.708	0.106	0.382
Myxoid liposarcoma	10	N/A	N/A	N/A	N/A	N/A	N/A	N/A	N/A	N/A	N/A
Well differentiated liposarcoma	4	N/A	N/A	N/A	N/A	N/A	0.362	N/A	0.808	0.362	0.362
Dedifferentiated liposarcoma	3	N/A	0.317	N/A	N/A	N/A	N/A	0.157	N/A	N/A	N/A
Ewing sarcoma	6	0.302	0.642	0.774	0.144	0.144	0.302	0.642	0.774	0.144	0.144
Malignant peripheral nerve sheath tumor	6	0.695	0.116	0.247	0.018	0.028	0.107	0.716	0.025	0.018	0.028
Angiosarcoma	6	0.110	0.583	0.896	0.583	0.060	0.388	0.705	0.117	0.705	0.066
Myxofibrosarcoma	6	0.025	0.157	0.134	N/A	0.025	0.025	0.018	0.091	N/A	0.025
Adult fibrosarcoma	5	0.039	0.886	0.445	0.351	0.199	0.207	0.364	0.299	0.774	0.782
Epithelioid sarcoma	4	N/A	0.362	0.362	0.083	0.193	N/A	0.918	0.918	0.083	0.218
Low grade myofibroblastic sarcoma	4	0.317	0.317	0.317	0.317	0.317	0.918	0.918	0.182	0.918	0.918
Alveolar rhabdomyosarcoma	3	N/A	N/A	0.225	0.157	0.225	N/A	N/A	0.225	0.157	0.225
Embryonal rhabdomyosarcoma	2	N/A	N/A	N/A	N/A	N/A	N/A	N/A	N/A	N/A	N/A
Pleomorphic rhabdomyosarcoma	2	0.317	0.317	0.317	0.317	0.317	0.317	0.317	0.317	0.317	0.317
Spindle cell/sclerosing rhabdomyosarcoma	1	N/A	N/A	N/A	N/A	N/A	N/A	N/A	N/A	N/A	N/A
Clear cell sarcoma	1	N/A	N/A	N/A	N/A	N/A	N/A	N/A	N/A	N/A	N/A

DSS; disease-specific survival, EFS; event-free survival, N/A; not available. *P* values less than 0.05 were considered significant and significant *P* values in this table are associated with shorter survival when PARP1, γH2AX, BRCA1, or BRCA2 are expressed.

**Fig 7:**
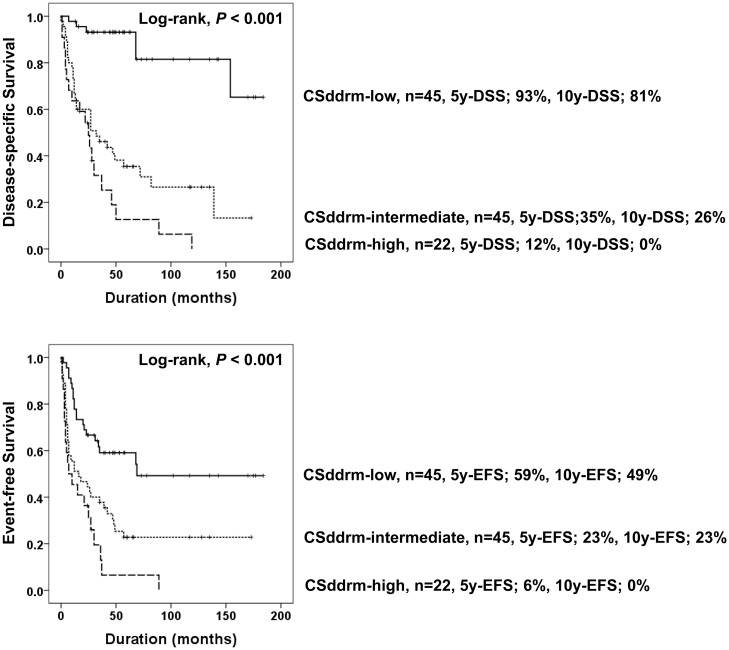
Kaplan-Meier survival analysis according to the combined expression pattern of PARP1, γH2AX, BRCA1, and BRCA2 in 112 soft tissue sarcomas. Soft tissue sarcomas were sub-grouped according to the combined score for the expression of PARP1, γH2AX, BRCA1, and BRCA2 (CSddrm). CSddrm was established with the sum of the positive scores for PARP1, γH2AX, BRCA1, and BRCA2 (negative; 0, positive; 1). CSddrm was grouped to CSddrm-low (CSddrm 0–1), CSddrm-intermediate (CSddrm 2–3), or CSddrm-high (CSddrm 4). 5y-DSS, disease-specific survival rate at five-years. 5-EFS, event-free survival rate at five-years. 10y-DSS, disease-specific survival rate at ten-years. 10y-EFS, event-free survival rate at ten-years.

Because the expression of PARP1, γH2AX, BRCA1, and BRCA2 were closely associated each other (Table 2) and their expression predicted shorter survival of STS patients (Table 3); we evaluated the prognostic significance of the combined expression patterns of PARP1, γH2AX, BRCA1, and BRCA2. When we divided STSs according to the positivity for these four markers, PARP1 expression was associated with shorter DSS in the γH2AX^-^, γH2AX^+^, BRCA1^-^, BRCA1^+^, BRCA2^-^, and BRCA2^+^ subgroups. γH2AX-positivity predicted shorter DSS and EFS in the BRCA1^-^, BRCA1^+^, BRCA2^-^, and BRCA2^+^ subgroups. BRCA2 expression was associated with shorter DSS in the PARP1^-^, PARP1^+^, γH2AX^-^, γH2AX^+^, BRCA2^-^, and BRCA2^+^ subgroups (Table 5). Because expression of these four markers is associated with DNA damage repair and our results also demonstrated significant correlation between their immunohistochemical expression, we evaluated the combined expression patterns of PARP1, γH2AX, BRCA1 and BRCA2. The positivity of each marker was scored zero or one (negative; 0, positive; 1) and a combined score derived by the number of positive markers. The ***c***ombined ***s***core for the immunohistochemical expression of ***D***NA ***d***amage ***r***esponse ***m***olecules PARP1, γH2AX, BRCA1, and BRCA2 (CSddrm, i.e., PARP1^+^/γH2AX^+^/BRCA1^+^/BRCA2^+^; 1+1+1+1 = CSddrm 4) ranged from zero (PARP1^-^/γH2AX^-^/BRCA1^-^/BRCA2^-^) to four (PARP1^+^/γH2AX^+^/BRCA1^+^/BRCA2^+^). Thereafter, CSddrm was grouped to CSddrm-low (CSddrm 0–1), CSddrm-intermediate (CSddrm 2–3), or CSddrm-high (CSddrm 4). In overall STS, CSddrm was significantly associated with shorter DSS and EFS (Table 3 and Fig 7). The DSS rates at ten-years (10y-DSS) of the CSddrm-low, CSddrm-intermediate, and CSddrm-high subgroups were 81%, 26%, and 0%, respectively. The EFS rates at ten-years (10y-EFS) of the CSddrm-low, the CSddrm-intermediate, and the CSddrm-high subgroups were 49%, 23%, and 0%, respectively (Fig 7).

**Table 5:** Univariate Cox proportional hazards regression analysis for survival in various subgroups of soft-tissue sarcomas according to the expression of BRCA1, BRCA2, PARP1, and γH2AX.

Subgroup			DSS				EFS			
			PARP1+ (*vs*. -)	γH2AX+ (vs. -)	BRCA2+ (vs. -)	BRCA1+ (vs. -)	PARP1+ (*vs*. -)	γH2AX+ (vs. -)	BRCA2+ (vs. -)	BRCA1+ (vs. -)
BRCA1	Negative	HR	4.097	6.438	4.017		1.516	2.791	2.264	
		(95% CI)	(1.649–10.177)	(2.338–17.727)	(1.708–9.449)		(0.785–2.925)	(1.421–5.483)	(1.169–4.383)	
		*P*	0.002	< 0.001	0.001		0.215	0.003	0.015	
	Positive	HR	5.544	3.332	4.41		3.939	2.726	2.552	
		(95% CI)	(1.900–16.175)	(1.437–7.728)	(1.645–11.826)		(1.606–9.663)	(1.271–5.845)	(1.114–5.847)	
		*P*	0.002	0.005	0.003		0.003	0.01	0.027	
BRCA2	Negative	HR	7.412	5.703		1.082	1.796	2.442		1.024
		(95% CI)	(2.005–27.400)	(1.737–18.727)		(0.346–3.383)	(0.843–3.829)	(1.144–5.216)		(0.431–2.428)
		*P*	0.003	0.004		0.892	0.129	0.021		0.958
	Positive	HR	2.511	2.98		1.23	1.955	2.148		1.164
		(95% CI)	(1.151–5.480)	(1.311–6.770)		(0.637–2.376)	(0.975–3.920)	(1.053–4.383)		(0.635–2.134)
		*P*	0.021	0.009		0.537	0.059	0.036		0.623
γH2AX	Negative	HR	7.797		3.728	2.618	1.664		1.985	1.216
		(95% CI)	(1.673–36.347)		(1.179–11.788)	(0.828–8.274)	(0.744–3.721)		(0.880–4.476)	(0.531–2.784)
		*P*	0.009		0.025	0.101	0.215		0.099	0.643
	Positive	HR	2.894		2.621	1.174	2.089		1.706	1.223
		(95% CI)	(1.384–6.051)		(1.250–5.496)	(0.627–2.196)	(1.099–3.969)		(0.903–3.222)	(0.696–2.151)
		*P*	0.005		0.011	0.616	0.024		0.1	0.484
PARP1	Negative	HR		6.349	4.839	1.311		2.23	2.049	0.642
		(95% CI)		(1.691–23.845)	(1.456–16.084)	(0.407–4.226)		(1.013–4.911)	(0.928–4.523)	(0.255–1.621)
		*P*		0.006	0.01	0.65		0.046	0.076	0.349
	Positive	HR		3.003	2.751	1.369		2.635	2.028	1.679
		(95% CI)		(1.433–6.295)	(1.304–5.801)	(0.727–2.579)		(1.354–5.125)	(1.067–3.856)	(0.925–3.049)
		*P*		0.004	0.008	0.331		0.004	0.031	0.088

Abbreviations: DSS; disease-specific survival, EFS; event-free survival, HR; hazard ratio, 95% CI; 95% confidence interval.

### The expression of PARP1, γH2AX, and BRCA2, and PARP1/γH2AX/BRCA1/BRCA2 expression pattern predicted shorter survival of STS patients by multivariate analysis

Multivariate analysis was performed with the factors significantly associated with DSS and/or EFS by univariate analysis. However, tumor differentiation, tumor necrosis, and depth of tumor were not included in multivariate analysis because these factors are a part of the WHO/FNCLCC tumor grade and tumor stage (Table 6). Among the 112 cases of STSs, age of patients, tumor stage, PARP1 expression, γH2AX-positivity, and CSddrm were independent prognostic predictor for both DSS and EFS (Table 6). The expression of PARP1 predicted a 3.181-fold greater risk of death and a 1.769-fold greater risk of relapse or death. Positive γH2AX expression predicted a 2.653-fold greater risk of death and a 2.061-fold greater risk of shorter EFS. The expression of BRCA2 was an independent predictor of shorter DSS.

**Table 6:** Multivariate Cox regression analysis for disease-specific survival and event-free survival in soft-tissue sarcoma patients.

Characteristics	DSS		EFS	
	HR (95% CI)	*P*	HR (95% CI)	*P*
Age,^a^ y, ≥ 60 (*vs* < 60)	2.064 (1.182–3.601)	0.011	1.902 (1.190–3.040)	0.007
Stage, ^a^ III & IV (*vs* I & II)	2.386 (1.222–4.658)	0.011	1.934 (1.172–3.190)	0.010
PARP1, ^a^ positive (*vs* negative)	3.181 (1.547–6.540)	0.002	1.769 (1.062–2.947)	0.028
γH2AX, ^a^ positive (*vs* negative)	2.653 (1.331–5.288)	0.006	2.061 (1.228–3.457)	0.006
BRCA2, ^a^ positive (*vs* negative)	1.996 (1.021–3.904)	0.043		
CSddrm,^b^ low (score 0–1)	1	< 0.001	1	0.009
intermediate (score 2–3)	7.272 (2.925–18.084)	< 0.001	2.127 (1.211–3.733)	0.009
high (score 4)	9.417 (3.476–25.509)	< 0.001	2.665 (1.353–5.250)	0.005

Abbreviations: DSS; disease-specific survival, EFS; event-free survival, HR; hazard ratio, 95% CI; 95% confidence interval, CSddrm; the combined score for the immunohistochemical expression of PARP1, γH2AX, BRCA1, and BRCA2.

^a^ The variables included in multivariate analysis were age, tumor stage, histological grade, mitotic count, and the expression of PARP1, γH2AX, BRCA1, and BRCA2.

^b^ The variables included in multivariate analysis were age, tumor stage, histological grade, mitotic count, and CSddrm.

## Discussion

Most chemotherapeutic regimens and radiation therapy, the most common treatments for human cancers, induce DNA damage and then damaged cancer cells undergo apoptosis when the damage is not repairable. However, if the damage induced by genotoxic therapeutic modalities is repaired by DDR molecules, the cancer cells survive [1, 3, 6]. Therefore, despite the tumor suppressive roles of the DDR molecules in repairing DNA damage to prevent the mutations needed for tumorigenesis, their expression might also result in the failure of first-line anti-cancer therapies [44]. Consistent with these reports, our results have demonstrated that the expression of DDR molecules PARP1, γH2AX, BRCA1, and BRCA2 were closely associated with each other and their expressions correlated with advanced clinicopathological factors, such as higher tumor stage, distant metastasis, and higher histologic grade. Especially, the expression of PARP1 and γH2AX were independent indicators of poor prognosis of both DSS and EFS of STS patients. In agreement with our results, it has been reported that the expression of PARP1 is associated with the progression of various human malignant tumors, such as gastric cancer [15], breast cancer [17], ovarian cancer [18], glioblastoma [19], and chordoma [45]. Among STSs, increased expression of PARP1 has been reported in Ewing sarcoma and the expression of PARP1 was upregulated by EWS-FLI1 fusion protein [46, 47]. In addition, our results demonstrated that the expression of PARP1 and γH2AX are significantly associated with shorter survival in the subpopulation of STS patients who received adjuvant chemotherapy and radiotherapy. These findings suggest that PARP1 and γH2AX might be involved in chemo- and radio-resistance. In agreement with this, a recent report has shown PARP1-mediated chemoresistance is associated with c-Myc mediated suppression of BIN1 [10]. However, even in the patients who did not received adjuvant chemotherapy or radiotherapy, the expression of PARP1 and γH2AX are significantly associated with shorter survival of STS patients (Figs 4 and 5). These findings suggest that therapy targeted at the PARP1/γH2AX pathway might be helpful for the treatment of STS patients because PARP1/γH2AX expression affects the survival of both STS patients who received adjuvant therapy and those that did not. In agreement with these findings, various PARP1 inhibitors have been shown to have anticancer effects in human cancers, including STS. Especially, suppression of PARP1 inhibited gastric cancer cells by inducing tumor suppressor FOXO3A [15]. Recently, the use of PARP1 inhibitors alone or in combination with other chemotherapeutic agents showed anti-tumor activity in rhabdomyosarcoma [32], malignant peripheral nerve sheath tumors [31] and Ewing sarcoma cells [24, 36, 37]. Therefore, when considering that the expression of PARP1 is very predictive in the prognosis of STS patients, a combination of PARP1 inhibitor and genotoxic cancer therapeutic modalities might be effective in the treatment of STS patients.

If a DNA SSB is not repairable and progresses to a DNA DSBs, H2AX becomes phosphorylated and recruits BRCA1/2, which is important for the repair of DNA DSBs. Therefore, the expression of γH2AX has been used as a sensitive marker of DNA DSBs [3, 5, 6, 44, 48]. However, paradoxically, the phosphorylation of H2AX during cancer therapy could confer a survival benefit to cancer cells [3, 6]. This study also showed that the expression of γH2AX was significantly associated with the expression of PARP1, BRCA1, and BRCA2 and an increased number of γH2AX foci, which were independent indicators of poor prognosis of STS patients. Consistent with our results, higher expression of endogenous γH2AX in various cancer cell lines, including fibrosarcoma and osteosarcoma cells, have been reported [49]. In addition, increased numbers of γH2AX expression foci was significantly associated with shorter survival of breast carcinoma [13, 14], endometrial carcinoma [21], and non-small cell lung cancer [50]. In prostatic cancer, higher expression of γH2AX was associated with chemoresistance by inducing G2/M arrest in cancer stem-like cells [51]. Thus, higher expression of γH2AX might be useful for the prediction of survival of STS patients and potentially a therapeutic target for STS patients [4, 6, 49].

A defects in *BRCA1/2* is one of the important causes of cancer development, especially of breast and ovarian carcinomas [7–9]. The risk of breast cancer by the age of 70 years has been reported as 57%-65% when there was *BRCA1* mutation and 45%-49% when there was *BRCA2* mutation [7, 8]. The risk of ovarian carcinoma at 70 years old was reported as 39% and 11% with *BRCA1* and *BRCA2* mutation, respectively [7]. However, the study for the BRCA-ness of STS is limited. One study has shown that 29% (25 of 85) of human uterine leiomyosarcomas are negative for BRCA1 immunohistochemical staining [12]. A search of the cBioPortal public database indicated that genetic alteration (mutation, deletion, or amplification) of *BRCA1* was seen in 0.5%-1% (1/207–2/240 cases) of STS and genetic alteration of *BRCA2* was seen in 3%-6% (6/207–14/240 cases) of STS [52, 53]. In addition, the Oncomine public database (search condition; *P* < 0.001, gene ranked in top 10%) indicated that higher expression of BRCA1 mRNA was seen in pleomorphic liposarcoma (n = 3 and n = 23), myxofibrosarcoma (n = 31), malignant fibrous histiocytoma (n = 9), fibrosarcoma (n = 7), leiomyosarcoma (n = 6), and round cell liposarcoma (n = 4) compared with normal tissue [48]. The expression of BRCA2 mRNA was higher in dedifferentiated liposarcoma (n = 46), myxofibrosarcoma (n = 31) leiomyosarcoma (n = 26), pleomorphic liposarcoma (n = 23), and malignant fibrous histiocytoma (n = 9) compared with normal tissue [48]. In the STS cases examined in this study, 55% and 49% of STS were classified as BRCA1-negative and BRCA2-negative subgroups, respectively. When we combine the data two public data bases and our results, the genetic status of *BRCA1/2* and expression of mRNA or protein of BRCA1/2 were inconsistent. The expression level of mRNA or protein of BRCA1/2 appeared higher despite a low incidence of genetic changes in *BRCA1/2*. However, the exact status of the BRCA-ness in STS is not clear due to the limited number of studies, and further study is needed. However, despite limitations for the estimation of the real functional status of BRCA1/2 in the cases of STS in this study, the expression of BRCA1 and BRCA2 were also significantly associated with progression and shorter survival of STS patients. These results are paradoxical to the tumor suppressive role of BRCA1/2 but consistent with the prognostic significance of the expression of PARP1 and γH2AX in STS. In line with the findings of this study, the expression of BRCA1/2 predicted shorter survival of breast carcinoma [14, 41] and ovarian carcinoma [16]. However, the studies based on BRCA1/2 immunostaining are limited in that the immunohistochemical expression of BRCA1/2 could not representatively reflect the genetic status of *BRCA1/2*. Nevertheless, a recent report has shown that immunohistochemical expression of BRCA1 could predict the genetic status of *BRCA1* in ovarian carcinoma [40]. In addition, it has been reported that the expression of BRCA1 is associated with platinum-resistance in ovarian carcinomas [16, 54] and chemoresistance in breast carcinomas [14]. Therefore, although further study is needed, these results suggest that BRCA1/2 may have a role in the progression and/or response to the therapy in STS.

In our previous studies on the prognostic markers of STS, individual expression of SIRT1, deleted breast cancer 1, β-catenin, programed death 1, and PD-L1 were independent indicators of poor prognosis for STS patients [29, 30]. The present study also shows that individual expression of PARP1, γH2AX, and BRCA2 are independent prognostic indicators of STSs. Moreover, an interesting finding of this study is that combined expression patterns of PARP1, γH2AX, BRCA1, and BRCA2 (CSddrm) were very predictive of the survival of STS patients. There were no live STS patients ten years after diagnosis which had tumors with a PARP1^+^/γH2AX^+^ /BRCA1^+^/BRCA2^+^ immunophenotype (CSddrm-high subgroup; 10-y DSS, 0%). In contrast, the DSS rate at ten-years in the CSddrm-low subgroup was 81%. Similarly, we previously reported on the prognostic significance of the combined expression patterns of the DDR molecules PARP1, γH2AX, BRCA1, and BRCA2 designated as CSbbph (***c***ombined ***s***core for the ***B***RCA1, ***B***RCA2, ***P***ARP1, and γ***H***2AX) in breast carcinoma [14]. Breast carcinoma in the CSbbph-low group that corresponds to a CSddrm-low immunophenotype has a 95% 10-year rate, but the ten-year DSS rate of breast carcinoma with a BRCA1^+^/BRCA2^+^/PARP1^+^/γH2AX^+^ immunophenotype was 35% [14]. These results suggest that the expression status of the DDR molecules PARP1, γH2AX, BRCA1, and BRCA2 are important for the prognosis of cancer patients and support the notion that these molecules might be therapeutic targets of STS. However, although we presented that the expression of PARP1, γH2AX, BRCA1, and BRCA2 are closely related with poor prognosis of STSs, this study is limited in that our cases are heterogeneous and a limited number of cases are included in each histologic subtype of STS. Therefore, further study is needed on the specific subtypes of STSs to clarify the role of DDR molecules in STSs. Especially, as we have shown in Table 4, when considering survival data in the subgroups of leiomyosarcoma and synovial sarcoma, further study in the larger subset of these subtypes of STS might be helpful.

In conclusion, this study demonstrates that the expression of the DDR molecules PARP1, γH2AX, BRCA1, and BRCA2 might be useful as prognostic indicators for STS patients. In addition, because the expression of DDR molecules are closely associated with clinical courses of STS patients, this study suggests the possibility that controlling the activity of DDR molecules might be a new possible therapeutic stratagem for the treatment of STS patients.
